# Identification of the replication region in pBCNF5603, a bacteriocin-encoding plasmid, in the enterotoxigenic *Clostridium perfringens* strain F5603

**DOI:** 10.1186/s12866-015-0443-3

**Published:** 2015-06-09

**Authors:** Kazuaki Miyamoto, Soshi Seike, Teruhisa Takagishi, Kensuke Okui, Masataka Oda, Masaya Takehara, Masahiro Nagahama

**Affiliations:** Department of Microbiology, Faculty of Pharmaceutical Sciences, Tokushima Bunri University, Yamashiro-cho 180, Tokushima, 770-8514 Japan; Division of Microbiology and Infectious Diseases, Niigata University Graduate School of Medical and Dental Sciences, 2-5274, Gakkocho-dori, Chuo-ku, Niigata 951-8514 Japan

**Keywords:** *Clostridium perfringens*, Bacteriocin gene, *rep* gene, Plasmid compatibility, Plasmid stability

## Abstract

**Background:**

Most recent studies of *Clostridium perfringens* plasmids have focused on toxin-encoding or antibiotic resistance plasmids. To cause intestinal disease, a toxigenic strain must grow in the intestines to levels allowing for sufficient toxin production and this in vivo growth often involves overcoming the normal intestinal microbial population. For this purpose, bacteriocin production might be important.

**Results:**

In this study, as the first step in the genetic analysis of a co-existing plasmid with an enterotoxin gene (*cpe*)-encoding plasmid, the bacteriocin gene-encoding plasmid, pBCNF5603, was completely sequenced. This plasmid has some homology with two previously sequenced *C. perfringens* plasmids, namely, pCP13 carrying a *cpb2* gene and pIP404 carrying a *bcn* gene. Using recombinant plasmids, the *rep* gene homologous to the PCP63 gene on pCP13 appeared to be functional. Comparative genomics indicated that the identified *rep* gene homologs were found on two additional toxin plasmids, pCP-OS1 and pCP-TS1. While functional analysis using recombinant plasmids indicated that pBCNF5603 and pCP13 are likely to be incompatible, the plasmid replication and partitioning region of pBCNF5603 alone was insufficient for stable maintenance of this plasmid.

**Conclusions:**

These findings suggest that pBCNF5603 evolved from recombination events between *C. perfringens* plasmids and inter-species mobile genetic element(s). In addition, the *bcn*-encoding plasmid, pBCNF5603, is likely to be included in the Inc family, which includes pCP13 and two variant *iota*-encoding plasmids. Furthermore, the *bcn* gene on pBCNF5603 could contribute to gastrointestinal disease induced by enterotoxigenic *C. perfringens*.

**Electronic supplementary material:**

The online version of this article (doi:10.1186/s12866-015-0443-3) contains supplementary material, which is available to authorized users.

## Background

*Clostridium perfringens* is an important Gram-positive pathogen, but also a member of the normal gastrointestinal flora of humans and animals [1]. The pathogenicity of this bacterium to humans and livestock largely depends upon toxin production, with 16 different *C. perfringens* toxins reported in the literature [1]. Most *C. perfringens* toxin genes, including three typing toxin (β, ε, ι) genes and recently identified novel toxin genes, such as necrotic enteritis B-like toxin gene (*netB*) and toxin *C. perfringens* large cytotoxin gene (*tepL*), are present on plasmids [2-4]. Therefore, the presence of toxin gene-encoding plasmids is crucial for *C. perfringens* virulence.

For *C. perfringens* strains producing *C. perfringens* enterotoxin (CPE) to cause non-foodborne human intestinal disease, they not only have to produce toxins, but also must overcome the normal microbial population so they can grow to pathogenic levels [5]. For this purpose, the production of bacteriocins might be useful [5]. Previous surveys of bacteriocins support this possibility since studies conducted in the 1960s and 1970s showed that many *C. perfringens* isolates produce bacteriocins [6]. However, a bacteriocin of *C. perfringens* has received much less attention recently than the toxins which induce the diseases caused by this bacterium, such as human and veterinary diarrhea.

On a completely sequenced *cpe*-encoding plasmid, pCPF5603, in the enterotoxigenic F5603 strain, which was isolated from a patient suffering from non-foodborne diarrheal disease, the gene encoding a bacteriocin that contributes to *C. perfringens* virulence was not identified. Therefore, an unidentified virulence factor such as a bacteriocin could be encoded on another co-existing plasmid.

In this study, the plasmid carrying a bacteriocin gene (*bcn*) homolog in the enterotoxigenic F5603 strain was completely sequenced. Thereafter, to understand the relationships with the co-existing prototype of toxin-encoding plasmid or other type(s) of toxin plasmid such as pCP13, the replication region of *bcn*-encoding plasmid was identified. Then, the replication region on pBCNF5603 was compared with those of other sequenced plasmids by comparative genomics because the replication region, which must contain the replication initiation protein (Rep) gene (*rep*), is a basic element of a plasmid [7]. Moreover, the identified replication and putative partitioning region was functionally investigated.

## Methods

### Bacterial strains and media

*Clostridium perfringens* strains F5603, NCTC8533, ATCC3624, and strain 13 were used in this study [8-10]. For the growth of these strains, TGY medium (3 % tryptic soy broth [TSB] [BD Bioscience, Tokyo, Japan], 2 % glucose, 1 % yeast extract [BD Bioscience, Tokyo, Japan], and 0.1 % L-cysteine [Wako Pure Chemical, Osaka, Japan]) with or without chloramphenicol (10 μg/ml) (Wako Pure Chemical, Osaka, Japan) was used as previously described [8, 9]. *E. coli* HB101 was used as the host for the recombinant plasmids used for DNA sequencing or for constructing recombinant plasmids carrying the putative replication region from *C. perfringens* plasmids. For the selection of *E. coli* or *C. perfringens* transformants, TSB agar containing ampicillin (100 μg/ml) (Wako Pure Chemical, Osaka, Japan) or chloramphenicol (30 μg/ml) and Brain Heart Infusion (BHI) (BD Bioscience, Tokyo, Japan) agar with chloramphenicol (15 μg/ml) were used, respectively.

### Complete sequencing of the bacteriocin-encoding plasmid pBCNF5603 in F5603 and plasmid pCP8533S12 carrying a pIP404-*rep* homolog in NCTC8533

Sequencing of a bacteriocin-encoding plasmid in strain F5603 was performed using a previously described method [8]. Briefly, crude plasmid DNA preparations were prepared as described previously [8] and then transformants carrying F5603 plasmid DNA fragments, which were not from the completely sequenced *cpe*-encoding plasmid pCPF5603, were selected and then sequenced. To complete the sequencing of pBCNF5603, long-range PCR analysis was performed using primers based upon end-sequence information. Those PCR products were then sequenced using a primer walking strategy.

For sequencing of a small plasmid named pCP8533S12, which is present in type B strain NCTC8533 and carries a pIP404 *rep* homolog, PCR primers were constructed based on the sequence of a small plasmid in ATCC3626, another type B strain (accession number: NZ_ABDV01000054). The PCR products obtained using those primers were then used for direct sequencing.

Putative ORFs on both sequenced plasmids were predicted with ORF finder software (http://www.ncbi.nml.nih.gov/gorf/gorf.html) and a putative Shine-Dalgarno sequence for each ORF was identified. Annotation of each ORF was predicted with BLAST blast.ncbi.nlm.nih.gov/Blast.egi web database software.

### Identification of the replication region in the bacteriocin-encoding plasmid pBCNF5603

Sequence analysis with BLAST indicated that pBCNF5603 mainly consists of two regions: a pCP13-like ORF region and a pIP404-like bacteriocin region (described in the results section). In addition, each region of pBCNF5603 was shown to harbor putative replication regions, namely, the PBCN16-18 region homologous to the PCP63-*parA* (*soj*)-*parB* (*spo0J*) gene cluster on pCP13 and the PBCN29-30 region homologous to the *cop*-*rep* region of pIP404. To investigate the replication ability of these two putative replication regions, each one was separately amplified by PCR. The primers used for amplification of the putative replication regions are listed in Additional file 1: Table S1. In these PCR reactions, each PCR mixture contained 1 μl of template DNA, 0.4 μl of GXL *Taq* polymerase (TAKARA BIO, Otsu, Japan), 3 μl of 2 mM NTPs, 10 μl of PCR buffer, and 2 μl of each primer pair (1 μM final concentration). PCR reactions were performed under conditions previously described [11]. After confirming PCR amplification, each PCR product was clarified using the QIAquick PCR purification kit (QIAGEN, Tokyo, Japan) and then digested with restriction enzymes: AvrII/BstBI (NEB, Tokyo, Japan) or AvrII/HpaI (NEB, Tokyo, Japan) for the pCP13-homologous putative replication region on pBCNF5603; SpeI/SnaBI (NEB, Tokyo, Japan) for the pIP404-homologous putative replication region on pBCNF5603; and BsmI/PflMI (NEB, Tokyo, Japan) for the putative *rep* region on pCP8533S12 (Fig. 2). Digested DNA fragments were subjected to filling with Klenow fragment polymerase (Promega, Tokyo, Japan), and were ligated into a fragment of shuttle vector pJIR750, in which the *rep* gene of pIP404 had been removed by digestion with XmnI (NEB, Tokyo, Japan) and SpeI (NEB, Tokyo, Japan) [12].

The resultant plasmids, named pKZ100, pKZ110, pKZ200, or pKZ210, were transformed into *E. coli* HB101 and then electroporated into transformable *C. perfringens* type A strains, ATCC3624 and strain 13, using Electro Cell Manipulator, ECM630 (BTX), according to the manufacturer’s instructions (Table 1) [13]. Briefly, 0.6 ml of overnight TGY culture of *C. perfringens* strain was inoculated into 20 ml of TGY medium and anaerobically incubated at 37 °C for 6 to 8 h. Bacterial cells were collected by centrifugation at 3,000 x g for 20 min. Collected cells were washed twice with SMP solution (270 mM sucrose [Pure Chemical, Osaka, Japan], 1 mM MgCl_2_ [Pure Chemical, Osaka, Japan] in 7 mM sodium phosphate solution, pH7.4) and then centrifuged. The washed bacterial pellet was re-suspended with 1.2 ml of SMP solution. A 600 μl aliquot of re-suspended cells was used for electroporation. Transformants carrying a recombinant plasmid were selected on BHI agar plates containing 15 μg/ml chloramphenicol.

**Table 1:** Plasmids constructed and used in this study

Plasmid	
pJIR750	HindIII/EcoRV (2629 bp) fragment of pIP404 replication region
pKZ100	pJIR750 (XmnI/SpeI) Ω pBCNF5603 PCR product (SpeI/SnaBI; 3120 bp) (*cop* and *rep* fragment)
pKZ110	pJIR750 (XmnI/SpeI) Ω pCP8533S12 PCR product (BsmI/PflMI; 2632 bp) (*rep* fragment)
pKZ200	pJIR750 (XmnI/SpeI) Ω pBCNF5603 PCR product (AvrII/BstBI; 5476 bp) (PCP63, *soj, parB* and PCP03 fragment)
pKZ210	pJIR750 (XmnI/SpeI) Ω pBCNF5603 PCR product (AvrII/HpaI; 2271 bp) (PCP63 fragment)

### Compatibility assay between recombinant plasmids derived from pBCNF5603 and pCP13

From the results of comparative genomics, the identified *rep* gene was highly similar to PCP63 (probable *rep* gene on pCP13) in strain 13. To understand the relationship between pBCNF5603 and pCP13, compatibility assay was performed. The constructed recombinant plasmid (pKZ200 or pKZ210) carrying the *rep* gene of pBCNF5603 was introduced into strain 13 according to the electroporation methods previously described [13]. In the final step of the electroporation procedures, electroporated samples were inoculated into 4 ml of pre-warmed TGY medium without antibiotics. After 3-h incubation at 37 °C, 0.2 ml of a 4 ml transformed culture sample was inoculated into 10 ml of TGY medium with chloramphenicol (10 mg/ml) and incubated at 37 °C. The next day, 0.2 ml of culture sample was inoculated in fresh TGY medium with chloramphenicol and incubated at 37 °C (passage 2). One-day culture of this second-passage sample was spread onto a BHI agar plate containing 15 mg/ml chloramphenicol, which was then anaerobically incubated at 37 °C.

To investigate the presence or absence of pCP13 in transformed *C. perfringens* colonies, randomly selected chloramphenicol-resistant colonies were tested by colony PCR assay for three genes: *cpb2* (the β2 toxin gene), *topA* (the gene of topoisomerase homolog), and *cna* (a putative collagen adhesion protein gene), on pCP13. For colony PCR reaction, colonies were suspended in 20 μl of distilled water and then heated at 100 °C for 10 min. After brief centrifugation, 1 μl of supernatant was used as a DNA template with PCR reaction. Each PCR mixture (50 μl) contained 1 μl of template DNA, 0.25 μl of EX *Taq* polymerase (TAKARA BIO, Otsu, Japan), 4 μl of 2.5 mM NTPs, 5 μl of PCR buffer, and 2 μl of each primer pair (1 μM final concentration). PCR reactions were performed under the following conditions: 95 °C, for 2 min; 35 cycles of 95 °C for 30 sec, 61 °C (for *cpb2*, *cna*) or 64 °C (for *topA*) for 30 sec, and 72 °C for 30 sec; and a single extension at 72 °C for 5 min. PCR products were electrophoresed on a 1.5 % agarose gel followed by staining with UltraPower™ DNA Stain (Gellex, Tokyo, Japan), and then fluorescence was detected using LAS-4000 (FUJIFILM).

### The stability of the constructed recombinant plasmid in two type A *C. perfringens* strains

Upstream of the *rep* gene, pBCNF5603 is also assumed to harbor the conserved plasmid partitioning region, including *parA* and *parB* genes and putative *parS* sequences. To investigate the contribution of putative plasmid partitioning regions to plasmid stability, the stability of a recombinant plasmid, pKZ200, which carries the replication and putative partitioning region, was estimated (Fig. 2). First, the recombinant plasmid was introduced into two transformable *C. perfringens* strains: ATCC3624 and strain 13. The type A ATCC3624 strain is likely to carry no plasmid, but strain 13 harbors pCP13, which carries a replication and partitioning region homologous to that of pBCNF5603, as described in the results section. Therefore, to estimate the stability of pKZ200 in strain 13, two chloramphenicol-resistant pCP13-free clones of strain 13-derived transformants were selected using the compatibility assay described above, and then used for plasmid stability assay; specifically, two clones of ATCC3624-derived transformants and two clones of pCP13-free strain 13 transformants were used.

Each of the stocked clones in cooked meat medium was inoculated into 10 ml of FTG medium (BD Bioscience, Tokyo, Japan) and incubated at 37 °C. Overnight cultures were then inoculated into 10 ml of TGY medium, incubated at 37 °C, then spread onto a BHI agar plate with chloramphenicol (15 mg/ml), and then anaerobically incubated at 37 °C. To estimate the stability of the recombinant plasmid, pKZ200, in *C. perfringens* strains, two separate colonies of each clone were inoculated into 10 ml of TGY medium cotaining chloramphenicol (10 μg/ml) and incubated at 37 °C. Then, 0.2 ml of overnight culture was inoculated into 10 ml of fresh TGY medium containing chloramphenicol. After incubation for 24 h at 37 °C, a 10 μl culture sample was transferred to 10 ml of fresh TGY without chloramphenicol, so that approximately 10 generations were obtained per growth cycle of 24 h (1:2^10^ dilution rate) [14]. These inoculated cultures were used as a first passage. Every 24 h, 10 μl of the full-grown cultures was transferred to 10 ml of fresh TGY. After 6 passages, the cultures were diluted and plated on a BHI plate without chloramphenicol. Determination of the fraction of recombinant plasmid cured cells in the population was performed by replica-picking of 25 randomly chosen colonies onto BHI with chloramphenicol (15 μg/ml) and without chloramphenicol, and scoring the chloramphenicol-sensitive colonies. To confirm the presence or absence of pKZ200, eight chloramphenicol-sensitive and eight chloramphenicol-resistant colonies were tested by colony PCR assay for the *rep* gene. Incompatibility between pKZ200 and indigenous pCP13 must influence the stability of the recombinant plasmid. To avoid the possibility of contamination with original strain 13, the same sixteen colonies were tested by *cpb2*-PCR assay.

## Results

### Organization of the *bcn5603*-encoding plasmid pBCNF5603 in F5603

**Fig. 1:**
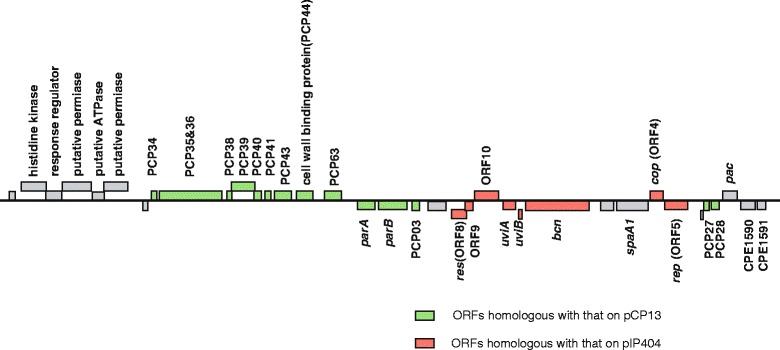
Genetic organization of predicted ORFs on pBCNF5603. Above the line, the orientation of putative ORFs is left to right, while under the line, it is the opposite. Green boxes depict the ORFs homologous to that on pCP13, and red boxes depict those homologous to that on pIP404. Sequences of pCP13 and pIP404 have been reported previously [15, 16]

In a previous study with pulsed-field gel electrophoresis (PFGE) analysis, *C. perfringens* F5603 strain carried two large plasmids, ~75 kb *cpe*-encoding plasmid and another ~40 kb plasmid [8] (Additional file 2: Figure S1). Sequencing revealed that pBCNF5603 (GenBank accession no. AB189671) is 36,695 bp in size, with 25.4 % GC content and encoding 36 ORFs. The size of sequenced plasmid closely agreed with the predicted size of a smaller ~40 kb plasmid in F5603 strain in PFGE analysis. In comparison with the sequences of pCP13 and pIP404, pBCNF5603 carries 15 or 8 ORFs that are highly homologous to ORFs present on pCP13 and pIP404, respectively [10, 15] (Fig. 1) (Additional file 1: Table S2). Of the fourteen pCP13 ORF homologs on pBCNF5603, a gene cluster from PBCN8 to PBCN15 was similarly arranged from PCP34 to PCP44, and a gene cluster from PBCN16 to PBCN19 was also from PCP63 to PCP03 (Fig. 1) (Additional file 1: Table S2) [10]. In the gene cluster from PBCN16 to PBCN19, pBCNF5603 carries *parA* and *parB* genes, which are highly homologous to the respective genes on pCP13, but which are absent from pIP404 [10, 15]. Therefore, this region might contain the genes for plasmid replication and maintenance, while the replication system of pCP13 has not yet been identified [10].

In eight shared ORFs with pIP404, pBCNF5603 encodes the putative bacteriocin BCN5603 gene (*bcn5603*) and upstream UV-inducible regulation genes, *uvi*A and *uvi*B, which are also present on pIP404 [16]. The BCN5 gene (*bcn*) was originally found in the type A human isolate CPN50. In comparative genomics, the product of the *bcn5603* gene, BCN5603, showed similarity to the gene products in *C. perfringens* strains: food poisoning strain-derived SM101, type E JGS1987, type C JGS1495, and type D JGS1721 strains, in addition to CPN50 strain. These bacteriocins in the virulent *C. perfringens* strain shared conserved domains: two SH3b domains (usually found on bacteriocins) and peptidase M14 superfamily domain, which contained two Zn-binding sites. In addition to these conserved domains on putative bacteriocins, BCN5603 carried putative cell wall binding repeat, and two glucan-binding repeats (Glucan65).

Of the bacteriocins in *C. perfringens*, the regulation of BCN5 production has been investigated most intensively; BCN5 is induced by an SOS response to UV light or mitomycin C treatment [16, 17]. Of the putative bacteriocin regulatory genes, the putative *uviA* gene on pBCNF5603 has 98.9 % nucleotide sequence homology with the *uviA* gene, which is the putative sigma factor for the *bcn5* gene on pIP404. In addition, on the basis of amino acid substitution of the *uviA* genes on both plasmids, regions 4.2 and 2.4 of the UviA sigma factor, which are implicated in interactions with the −35 and −10 sequence of the promoter, were completely identical [16]. The *uviB* gene on pBCNF5603 had a completely identical nucleotide sequence with the *uviB* gene, which is the putative bacteriocin immunity-related gene on pIP404 [16]. Moreover, the *bcn5603*-*uviA*-*uviB* gene cluster had three promoter sequences almost identical to those in the original *bcn* gene region on pIP404. Specifically, compared with the matching promoter sequences of BCN5 [17], the *bcn5603* gene has two putative promoter sequences: P1 (the strongest promoter of the *bcn* gene on pIP404) and P2, which are completely identical, as well as P3 with only one nucleotide change. Therefore, the *bcn5603* gene might be dependent on UviA for activation. Furthermore, the P4 and P5 promoters upstream of the *uviA* gene on pBCNF5603 had identical sequences to those on pIP404, and the putative repressor LexA-binding site of the *bcn5603* gene also had an identical sequence with the corresponding site in the pIP404 BCN5 regulatory region [17]. From these highly similar sequences in the regulatory region, the enterotoxigenic strain F5603 might produce BCN5603 in a similar manner to BCN5 by strain CNP50.

pBCNF5603 also carries a cluster of five ORFs (putatively encoding a sensor histidine kinase, response regulator, ATPase, and two permease genes) with high homology to respective genes present on the chromosome of *E. faecalis* V583 and the *S. agalactiae* 2603V/R strain [18, 19] (Fig. 1) (Additional file 1: Table S2).

Overall, pBCNF5603 was identified as a bacteriocin-encoding plasmid that developed from a mosaic fusion of *C. perfringens* plasmids, *cpb2*-encoding pCP13 and *bcn*-encoding pIP404, and a foreign gene cluster.

### Identification of the replication region of pBCNF5603

Completely sequenced pBCNF5603 is likely to be an important plasmid for *C. perfringens* virulence because this plasmid encodes a bacteriocin gene. Moreover, pBCNF5603 co-exists with one of the prototype *cpe*-encoding plasmids, pCPF5603. Therefore, to investigate further the bacteriocin gene-encoding plasmid, the replication region of pBCNF5603 was initially identified in the current study. Sequencing and comparative genomics showed that pBCNF5603 carried two sets of potential plasmid replication regions, namely, the region containing putative plasmid partitioning genes, *parA* and *parB*, which was similar to that region on pCP13 (Fig. 1), and putative *rep* and *cop* genes similar to the replication region of pIP404.

**Fig. 2:**
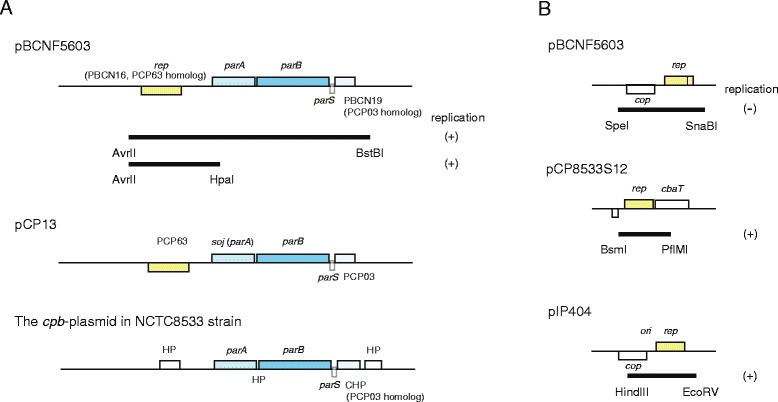
**a** Schematic comparison of the newly identified replication and partitioning region on: I) *C. perfringens* plasmid pBCNF5603 of F5603, II) *C. perfringens* plasmid pCP13 of strain 13, or III) *C. perfringens* plasmid pCP8533cpb of NCTC8533. The yellow boxes show ORFs on the putative *rep* gene, and blue boxes show conserved genes in the putative partitioning region; the smallest boxes show the region of a putative *parS* sequence. HP depicts the genes encoding a hypothetical protein, and CHP depicts the gene encoding a conserved hypothetical protein. Shaded bars show the region inserted in pKZ200 (upper bar) or pKZ210 (lower bar). **b** Schematic comparison of the putative replication region on: I) *C. perfringens* plasmid pBCNF5603 of F5603, II) *C. perfringens* plasmid pCP8533S12 of NCTC8533. The yellow boxes show the putative *rep* genes, and orange box shows a putative deleted region of a putative *rep* gene on pBCNF5603, which is homologous to the *rep* gene on pIP404 or on pCP8533S12, or III) *C. perfringens* plasmid pIP404 of CPN50

**Fig. 3:**
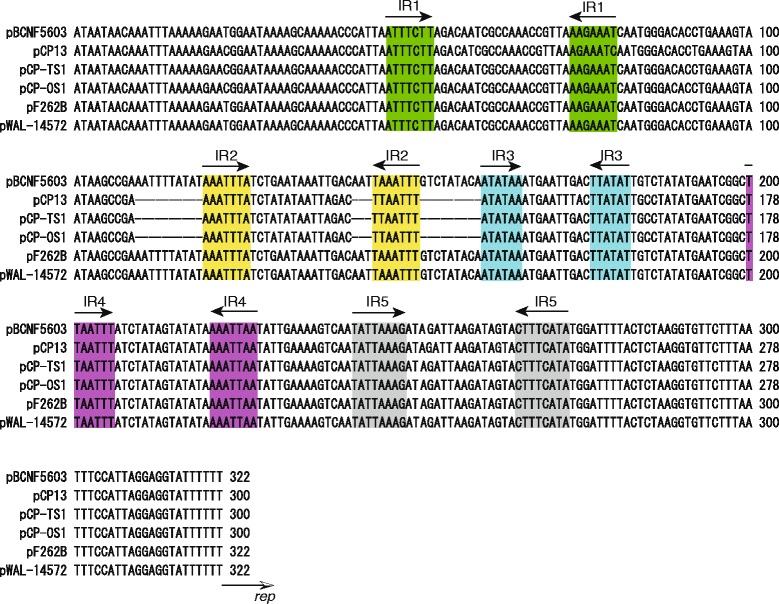
Repeated elements in the upstream region of the identified *rep* genes on pBCNF5603 and of the PCP63 homologs of other plasmids. The arrow-headed bars depict the five pairs of inverted repeats (IR1 to IR5). The *rep* gene ORF starts next to the final nucleotide

It was initially shown that a recombinant plasmid named pKZ200 carrying a ~5.5 kb fragment including the PBCN16 (PCP63 homolog)-*parA*-*parB*-PBCN19 (PCP03 homolog) gene cluster could replicate in *C. perfringens* strains, ATCC3624 and strain 13 (Fig. 2a, Table 1). The recombinant plasmid that still supported plasmid replication in two strains was pKZ210, carrying a smaller ~2.3 kb region containing PBCN16, and its upstream region (including the partial *parA* ORF) (Fig. 2a, Table 1). These results indicate that PBCN16, the product of which is a homolog of the PCP63 product, was likely to be the *rep* gene of pBCNF5603. The upstream region of the identified *rep* gene, five distinct pairs of 6 to 8 bp inverted repeats (IRs), which could act as iteron-like sequences in the initiation of pBCNF5603 replication, and an AT-rich region, which is also important for plasmid replication, were identified (Fig. 3) [7]. Therefore, pBCNF5603 was identified as one of the iteron-containing plasmids (ICP) in *C. perfringens*.

Concerning the replication ability of the second region, ATCC3624 strain carrying the constructed plasmid named pKZ100, which carried the putative *cop*-*rep* region similar to the replication region of pIP404, was not detected in several electroporation experiments (Table 1, Fig. 2b). On the other hand, a constructed plasmid named pKZ110 carrying the ~2.6 kb region containing the putative *rep* gene of pCP8533S12 could replicate in the ATCC3624 strain (Table 1, Fig. 2b).

Collectively, only the region containing genes similar to the genes on pCP13 was identified as a region carrying the newly identified *rep* gene, while two potential replication (and maintenance) regions were identified on pBCNF5603 by comparative genomics.

### Comparative genomic analysis of the genes in the identified replication region

By comparative genomics using the GenBank database, plasmids carrying a homolog of the identified *rep* gene of pBCNF5603 were investigated. In addition to PCP63 on pCP13 (accession number: AP003515), four other homologous genes were found, namely, a gene on pCP-OS1 (accession number: AP013033), a gene on pCP-TS1 (accession number: AP013034) (these two plasmids harbor the recently identified iota toxin gene variants), a gene on pF262B (accession number: AFES01000051), and a gene on a plasmid in the WAL-14572 strain (accession number: ADLP01000024) [20, 21] (Table 2). These genes encode hypothetical proteins. Of these genes, PCP63 (651 bp) on pCP13 was the smallest; the *rep* gene on pBCNF5603 was 852 bp long, and the homologous genes on pCP-TS1 or pCP-OS1 and those of pF262B or plasmid in WAL-14572 were 918 bp and 906 bp, respectively. In the upstream region, each of the putative *rep* genes on the four plasmids carried iteron-like sequences that were highly similar to those of the *rep* gene of pBCNF5603, while the putative iteron sequences were not identified in the upstream region of PCP63 (Fig. 3). Moreover, pBCNF5603 and pCP13 in strain 13 from our laboratory stocks were likely to be incompatible (described in the results section). Therefore, for further comparison of those homologs, sequencing analysis of the putative replication region of pCP13 in strain 13 in our laboratory stocks was performed. In terms of the sequencing results of the PCP63 region in strain 13 from our laboratory stocks, PCP63 was 906 bp long and also harbored highly similar iteron-like sequences with a putative *rep* gene on other plasmids (accession number: LC011526) (Fig. 3, Table 2).

**Table 2:** Similarity of the genes on the replication and partitioning region among C. perfringens plasmids carrying PCP63 homolog

	PCP63 homologs		*parA* (*soj*)		*parB*		*parS* sequence	PCP03 homologs	
	ORF size	Identity^a^	ORF size	Identity^a^	ORF size	Identity^a^	length	ORF size	Identity^a^
pBCNF5603	852 bp	100 %	753 bp	100 %	1278 bp	100 %	117 bp	363 bp	100 %
pCP13	906 bp	92 %	753 bp	99 %	1281 bp	96 %	117 bp	363 bp	94 %
pCP-TS1	918 bp	91 %	753 bp	97 %	1281 bp	99 %	117 bp	363 bp	90 %
pCP-OS1	918 bp	90 %	753 bp	97 %	1281 bp	99 %	117 bp	363 bp	90 %
pF262B	906 bp	93 %	753 bp	99 %	1278 bp	100 %	117 bp	363 bp	99 %
pWAL-14572	906 bp	93 %	753 bp	99 %	1278 bp	100 %	117 bp	363 bp	99 %
pCP8533cpb	-	-	753 bp	98 %	1281 bp	96 %	117 bp	363 bp	96 %
JGS1945cpb	-	-	753 bp	99 %	1281 bp	96 %	117 bp	363 bp	95 %

^a^: Substitutional amino acid identity

Collectively, bacteriocin- or toxin-encoding plasmids harbor the *rep* gene homologous to that of pBCNF5603; the plasmid group carrying the pBCNF5603-type *rep* gene is likely to contribute to *C. perfringens* virulence.

Sequencing also showed that pBCNF5603 carries several gene homologs that appear to be involved in the replication and maintenance systems of pIP404 [15]. The replication system of pIP404 is similar to the Inc18 family of plasmids, whose replication system consists of *cop*-*rep*-*ori*-*res* genes [15]. Of these four replication component genes, pBCNF5603 was found to carry two ORFs, namely, PBCN29 and PBCN30, which are highly similar to pIP404 genes that are thought to encode putative plasmid stability and replication factors, respectively [15]. Therefore, a candidate Rep protein of pBCNF5603 is probably encoded by PBCN30, which is homologous (92 % identity) to the *rep* gene (ORF5) of pIP404 (Additional file 1: Table S1). However, PBCN30, which is ~1,000 bp in size, is smaller than pIP404 ORF5 (~1,200 bp in length) by approximately 200 bp in the 3’ region (Fig. 2b).

A putative *rep* gene, which is also similar to the *rep* gene on pIP404, was also found on a small plasmid (~12 kb) in type B strain NCTC8533 and named pCP8533S12 (accession number: AB736082, Additional file 1: Table S3) (Fig. 2b). The size of this *rep* gene was similar to that of the *rep* gene on pIP404, but larger than that of the PBCN30 gene on pBCN5603. In the GenBank database, homologous *rep* genes were found on a ~12 kb plasmid in type B strain ATCC3626 (accession number: ABDV01000054), on a ~13 kb plasmid in type C strain JGS1945 (accession number: ABDU01000032), on a ~4 kb plasmid in type D strain JGS1721 (accession number: ABOO01000094), and on a ~12 kb bacteriocin-encoding plasmid in type A enterotoxigenic SM101 strains (accession number: NC_008264). The length of these putative *rep* genes was ~1,200 bp, while that of a pIP404-*rep* homologous gene (PBCN30) on pBCNF5603 was smaller than other putative *rep* genes by ~1,000 bp, with truncation at the 3’ end because of an insertion (deletion) producing a stop codon at a later site. Interestingly, a ~1,000 bp sequence at the 5’ end of these putative *rep* genes was very similar (percent identities of nucleotides among the *rep* genes of more than 90 %; ranging from 91.4 to 98.0 %), while ~200 bp sequences on the 3’ ends of these genes were less conserved (percent identities of 57.9-84.7 %).

### Compatibility between pCP13 and constructed recombinant plasmids carrying the identified replication region

Each set of twenty-five chloramphenicol-resistant colonies from second-passaged cultures of strain 13, which were introduced by recombinant plasmids, pKZ210 (carrying PCP63 homolog and its upstream region) or pKZ200 (carrying the *parA* and *parB* genes, and putative *parS* region), were investigated (Fig. 2a). No chloramphenicol-resistant colonies showed a positive reaction in the PCR assay for the *cpb2* gene, and these investigated colonies also showed a negative reaction in the PCR assay for the *topA* gene or the *cna* gene on pCP13; this was likely to have occurred to cure the *cpb2*-encoding plasmid, pCP13, in strain 13-derived colonies introduced by one of the recombinant plasmids that carry the identified replication region with or without a putative partitioning region.

### Stability of the recombinant plasmid carrying the identified replication region and a putative partitioning region

The putative partitioning region of pBCNF5603 contains two genes, *parA* (a putative ATPase gene) and *parB* (centromere-binding protein gene), and a putative *parS* sequence (Fig. 2a). The *parA* and *parB* genes were found on five other plasmids that harbor the identified *rep* gene homolog, and those gene products had more than 96 % identity in terms of substituted amino acids (Table 2). A putative *parS* sequence contained IR1, IR2, and one repeated sequence (Additional file 3: Figure S2), and the sequences of the *parS* region were almost identical among six plasmids; that is, these plasmids are likely to carry a similar plasmid replication and partitioning region to that of pBCNF5603. These findings also indicate that a putative partitioning system on these plasmids might be the *parABS* system.

To investigate the contribution of the putative partitioning system on pBCNF5603 to plasmid stability, the fraction carrying the recombinant plasmid, pKZ200, was monitored without any selection in two different *C. perfringens* hosts. In transformants derived from *C. perfringens* strains, ATCC3624 and strain 13, the proportion of chloramphenicol-resistant colonies dropped to ~40 % after 60 generations without antibiotic selection, while it was more than 80 % under antibiotic pressure (Table 3). Therefore, the recombinant plasmid was considered to be unstable in both strains; all transformants from both strains showed high plasmid loss in terms of the proportion of plasmid-free cells. Moreover, there was no clear relationship between the host strain type and the stability of the recombinant plasmid, while strain 13 originally harbored an indigenous pCP13 that carries the closely related replication and partioning region of pBCNF5603.

**Table 3:** Stability of a recombinant plasmid, pKZ200, and plasmids, pBCNF5603 and pCP13, in C. perfringens strains

Incidence of colonies carrying a recombinant plasmid, pKZ200, in ATCC3624 strain		
		Rate of Cp resistant colonies^*^
clone 1	Culture with Cp	92 %
	Culture without Cp	40 %
clone 2	Culture with Cp	84 %
	Culture without Cp	44 %
Incidence of colonies carrying a ecombinant plasmid, pKZ200, in strain 13		
		Rate of Cp resistant colonies^*^
clone 1	Culture with Cp	80 %
	Culture without Cp	40 %
clone 2	Culture with Cp	96 %
	Culture without Cp	44 %
Incidence of colonies carrying a bacteriosin gene (*bcn5603*)-encoding plasmid, pBCNF5603, in F5603 strain		
		Rate of PCR positive colonies^*, #^
clone 1	PCR for the rep gene	100 %
	PCR for the bcn5603 gene	100 %
clone 2	PCR for the rep gene	100 %
	PCR for the bcn5603 gene	100 %
Incidence of colonies carrying the beta2 toxin gene-encoding plasmid, pCP13, in strain 13		
		Rate of PCR positive colonies^*, #^
clone 1	PCR for the *PCP63* gene	100 %
	PCR for the *cpb2* gene	100 %
clone 2	PCR for the *PCP63* gene	100 %
	PCR for the *cpb2* gene	100 %

Culture samples with or without 10 µg/ml chloramphenicol were collected after ~60 generations

^*^: Rate of randomly selected colonies

^#^: To investigate the presence of pBCNF5603 in F5603 strain or pCP13 in strain13, randomly selected colonies were tested with colony PCR assay for three genes, *rep* (PCP63), *bcn5603*, and *cpb2* genes. Colony PCR reactions were performed under the same conditions as described in the plasmid compatibility section

In contrast to the stability of the recombinant plasmid in *C. perfringens* strains, an indigenous plasmid in the original strain, pBCNF5603 in F5603 or pCP13 in strain 13, was extremely stable; after 60 generations, no indigenous plasmid-cured colonies were detected by PCR assays for two genes: the *rep* and the *bcn5603* or *cpb2* genes, respectively (Table 3).

## Discussion

Bacteriocins could contribute to diseases caused by *C. perfringens*, for example, promoting intestinal colonization [5]. Of several identified *C. perfringens* bacteriocins, the most investigated one is BCN5 in the type A *cpe*-negative strain CPN50. BCN5 production is induced by stress, such as UV irradiation, and its gene is encoded by the well-studied plasmid pIP404, which has been completely sequenced and for which the gene for plasmid replication was identified [15]. From the results in the current study, a plasmid named pBCNF5603, which does not encode toxins, was found to co-exist with the *cpe*/*cpb2* toxin gene encoding plasmid pCPF5603 in the enterotoxigenic type A strain F5603. pBCNF5603 carries the putative bacteriocin gene *bcn5603* and its regulatory sequences, as determined from the results of complete sequence analysis (Fig. 1) (Additional file 1: Table S2). Of all *C. perfringens* bacteriocin genes, only the genetic background of the bacteriocin (BCN5) gene (*bcn*) found on pIP404 in the type A CPN50 strain has been carefully investigated [16, 17]. The same as the *bcn* gene on pIP404, the *bcn5603* gene had regulatory *uviA* and *uviB* genes and regulatory sequences, such as promoter sequences and the LexA-binding sequence. Moreover, these regulatory sequences were almost identical to the respective sequences on pIP404. In fact, *C. perfringens* F5603 strain was likely to produce a bacteriocin, which was promoted by UV irradiation, using pCP13-cured strain 13 as an indicator strain. Moreover, transcription of the *bcn5603* gene was likely to be promoted by the addition of mitomycin C (data not shown); that is, the extreme conservation of the *bcn* gene cluster on pBCNF5606 indicates that production of the BCN5603 bacteriocin might be induced in the same manner as for BCN5 in the CPN50 strain.

Comparative genomics using the GenBank database indicated that *bcn5603* gene homologs have been found in several other virulent strains. These homologs encode putative bacteriocins that carry commonly conserved domains, namely, SH3 and peptidase M13 family domains. Moreover, a recent PCR survey indicated that BCN5 gene homologs are found in *cpe*-positive type A human disease isolates (F4013 and NCTC8798), as well as type B, D, and E animal disease isolates [22]. Taking these findings together, the production of this group of bacteriocins by *C. perfringens* virulent strains might benefit for them compared with other enteric bacteria in the human or animal gut, so this group of bacteriocins might contribute to the virulence of *C. perfringens* strains.

To date, only two kinds of *rep* gene have been identified on *C. perfringens* plasmids: the *rep* gene on the antibiotic resistance pCW3 plasmid and the *rep* gene on the bacteriocin gene-encoding pIP404 [15, 23]. The *rep* gene of the most representative toxin gene-encoding plasmids is highly similar to the *rep* gene of pCW3 [8, 23]. To understand the relationship between pBCNF5603 and other plasmids, especially plasmids carrying the previously identified *rep* genes, the replication region of pBCNF5603 was identified. On the basis of sequence information for pBCNF5603, a candidate of the putative replication region, which is similar to the putative pCP13 plasmid replication region, was identified, while the *rep* gene of pCP13 has not yet been identified. The results of investigating the replication ability of the PBCN16 region indicated that recombinant plasmids (pKZ200 and pKZ210), both of which carry PBCN16 and its upstream region, were replicable in two transformable *C. perfringens* strains, ATCC3624 and strain 13; the similarly organized region of pBCNF5603 with that of pCP13 is most likely to be the replication region that must contain the *rep* gene. Moreover, five inverted repeated sequences, probably iterons, and an AT-rich region were also identified upstream of the PBCN16 ORF; PBCN16 is likely to be the *rep* gene of pBCNF5603, and therefore pBCNF5603 might be an iteron-containing plasmid (ICP). By comparative genomics using the GenBank database, PBCN16 homologs were found in several other plasmids including pCP13, pCP-OS1, and pCP-TS1, which are toxin-encoding plasmids other than the prototypes of toxin-encoding plasmid [20]. Moreover, these plasmids carry upstream iteron-like sequences that are highly similar to those sequences of PBCN16 (Fig. 3). Therefore, the plasmid family that carries the PBCN16 homolog in the putative replication region is also likely to be ICP.

Prototypes of toxin-encoding plasmids carry the common replication and partitioning system, the *parMRC* system, and these systems might be related to plasmid compatibility [23, 24]. In the same *C. perfringens* cells of the F5603 strain, the bacteriocin-encoding plasmid pBCNF5603, which carries a different replication and partitioning system, the *parABS* system, can co-exist with one of the prototypes of toxin-encoding plasmids, *cpe*-encoding plasmid pCPF5603. On the other hand, recombinant plasmids carrying the identified *rep* gene and upstream iteron sequences of pBCNF5603 were likely to induce pCP13 curing in strain 13; pBCNF5603 and pCP13 might be incompatible plasmids, possibly because of iteron-based incompatibility. In general, two components, Rep and iterons, lie at the center of all models of ICP replication control, so replication-mediated incompatibility has been used to classify plasmids [7]. Taking these findings together, pBCNF5603 and pCP13 are likely to be classified into the same Inc group, but different groups from the prototypes of toxin-encoding plasmid. Moreover, on the basis of the high similarity of upstream iteron sequences of the putative *rep* gene, recently sequenced plasmids, pCP-TS1, pCP-OS1, and other plasmids (pF262B and plasmid in WAL-14572 strain), are likely to belong to the pBCNF5603 Inc family [20, 21].

A recombinant plasmid named pKZ100, which carries pIP404 *rep* homolog, PBCN30, is non-functional in *C. perfringens* strain, while PBCN30 was also identified as a candidate of the *rep* gene on pBCNF5603 by a BLAST search (Fig. 2b). On the other hand, pIP404 *rep* gene homolog on a small plasmid, pCP8533S12, in type B strain NCTC8533 is functional based on the transformation experiment results obtained using another recombinant plasmid, pKZ110. By comparative genomics using the GenBank database, pIP404 *rep* gene homologs were also found in several strains that also harbor prototypes of toxin-encoding plasmid(s). Comparing the sequence of these *rep* homologs in different strains, PBCN30 on pBCNF5603 was ~200 bp smaller than other homologs; in addition, PBCN30 on pBCNF5603 has a point mutation and, as a result, may have become non-functional (Fig. 2b). From these findings, it was suggested that ~200 bp on the 3’ side of this type of *rep* gene might be important for plasmid replication.

Upstream of the PBCN16 *rep* gene, the putative plasmid partitioning-related genes (*parA*, *parB*, and maybe PCP03 homolog) and a putative *parS* sequence were identified (Fig. 2a). A similarly organized putative partitioning region was also found on pCP13, pCP-OS1, pCP-TS1, pF262, and plasmid in the WAL-14572 strain. Therefore, the highly homologous *rep* gene (more than 90 % identity) and a putative partitioning region are likely to be a core region of these plasmids. In general, the partitioning region has a central role in plasmid segregation and is thus important for plasmid stability [25]. To investigate the contribution of the putative partitioning region of pBCNF5603 to plasmid stability, the stability of the recombinant plasmid that carries the replication and partitioning region was evaluated in two electro-transformable strains, ATCC3624 and strain 13. Strain 13 originally carries the indigenous plasmid, pCP13, which has a replication and partitioning region that is closely related to that of pBCNF5603, while the ATCC3624 strain naturally harbors no plasmid. Although strain 13 and F5603 strain stably carried pCP13 or pBCNF5603, respectively, the recombinant plasmid that carries the pBCNF5603-derived replication and partitioning region was relatively unstable, even in pCP13-free strain 13-derived strain, as in the ATCC3624 strain. These findings might indicate that the identified replication and partitioning region of pBCNF5603 is insufficient for plasmid stability, while the replication and partitioning region is important for plasmid stability [25]. The possible reasons for recombinant plasmid instability are as follows: 1) the influence of the integrity of iteron sequences and arrangement; 2) the sequence of the genes in this region might be strictly controlled: the integrity of these regions might be important for plasmid stability; and 3) the gene(s) encoding hypothetical or conserved hypothetical protein on pBCNF5603 could have an important role in the stability of the plasmid. In addition to these possibilities, host factors could possibly promote plasmid stability; the replication and partitioning region might co-operate with host chromosomal factors [25]. Strain 13 is likely to have a chromosomal genetic background that is more similar to the F5603 strain than to the ATCC3624 strain, given previous MLST results [22]. However, the stability levels of the recombinant plasmid that carries the replication and partitioning region were very similar in those two *C. perfringens* strains. Therefore, the contribution of the host factors to the stability of the recombinant plasmid could differ compared with plasmids in *E. coli*.

Interestingly, a similarly organized putative plasmid partitioning region, the *parABS* region, was found on a β toxin gene (*cpb*)-encoding plasmid, pCP8533cbp, in the type B NCTC8533 strain (accession number: AB736083) (Fig. 2a). By comparative genomics using the GenBank database, these conserved *parABS* regions were also identified on the *cpb* plasmids in the type B ATCC3626 strain (accession number: NZ_ABDV01000024) and in the type C JGS1945 strain (accession number: NZ_ABDU01000064). However, these *cpb*-encoding plasmids did not harbor a PCP63 homolog on the upstream region of the *parA* gene. The *parABS* region on these *cpb*-encoding plasmids could be related to plasmid properties, such as partitioning element-based plasmid incompatibility.

To our knowledge, this is the first report about identification of the *rep* gene of a bacteriocin-encoding plasmid in enterotoxigenic *C. perfringens* strain. The *rep* gene identified in the current study is unique and might be encoded by important plasmids because the identified *rep* gene homologs were found on not only the bacteriocin-encoding plasmid, but also toxin-encoding plasmids. In addition, *C. perfringens* plasmids that carry the identified *rep* gene or its homolog might be the same Inc family plasmids.

## Conclusions

The current study demonstrated that i) the replication region of bacteriocin gene encoding plasmid, pBCNF5603 in enterotoxigenic *C. perfringens* strain was identified, ii) the plasmid carrying the identified *rep* gene are predicted as a iteron-containing plasmid (ICP), iii) the identified replication region are also found on toxin gene encoding plasmid in virulent strains, e.g. BEC (binary enterotoxin of *C. perfringens*) gene encoding plasmids, iv) the identified replication region contributes plasmid compatibility; plasmids carrying the homologs with the identified *rep* gene should belong to the same Inc family, v) the plasmid replication and the predicted plasmid partitioning regions are insufficient for stable maintenance of recombinant plasmid which carry those of pBCNF5603 in *C. perfringens* strains.

Collectively, findings in the current study provide the important insights of the bacteriocin gene and toxin gene encoding plasmids in virulent *C. perfringens* strains; contribution of the replication region to plasmid compatibility have not been investigated in previous studies, while the replication region of a few plasmids in virulent *C. perfringens* strains was reported. Moreover, role of the plasmid replication and partitioning regions on *C. perfringens* plasmid stability have not been investigated, although the important plasmids are likely to be highly stable in virulent strains. Further detailed studies on the issues investigated in the current study should help in understanding plasmid biology in virulent *C. perfringens* strains.

## Additional files

Additional file 1: Table S1. PCR primers for amplification of putative replication regions and used for the detection of genes with colony PCR assays. **Table S2.**. Homology search results of putative ORFs on pBCNF5603. **Table S3.** Homology search results of putative ORFs on pCP8533S12.

Additional file 2: Figure S1. PFGE analysis of plasmids from *C. perfringens* F5603 strain. Agarose plug containing genomic DNA from F5603 strains was subjected to PFGE and stained with ethidium bromide (Wako Pure Chemical, Osaka, Japan). M: Low Range PFG marker (NEB, Tokyo, Japan).

Additional file 3: Figure S2. Repeated elements on the putative parS region. The arrow-headed bars depict the two pairs of inverted repeats (IR1 and IR2). And green colored regions depict two short-repeated sequences.
