# Supplementary figures and images for: Identification of the replication region in pBCNF5603, a bacteriocin-encoding plasmid, in the enterotoxigenic Clostridium perfringens strain F5603
Source: BMC Microbiol. 2015 Jun 9;15:118. doi: 10.1186/s12866-015-0443-3 (PMC4459074; doi:10.1186/s12866-015-0443-3)

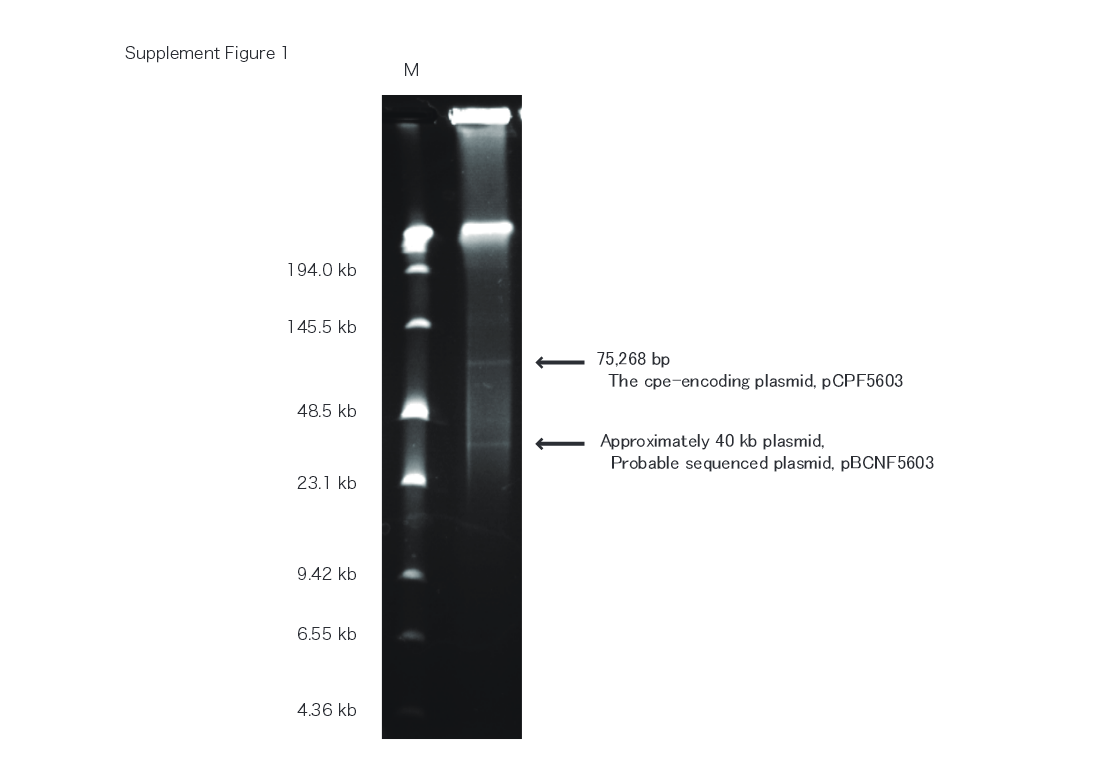

Supplement: Supplementary file 2 — PFGE analysis of plasmids from C. perfringens F5603 strain. Agarose plug containing genomic DNA from F5603 strains was subjected to PFGE and stained with ethidium bromide (Wako Pure Chemical, Osaka, Japan). M: Low Range PFG marker (NEB, Tokyo, Japan). [file 12866_2015_443_MOESM2_ESM.png]

Supplementary Figure 2

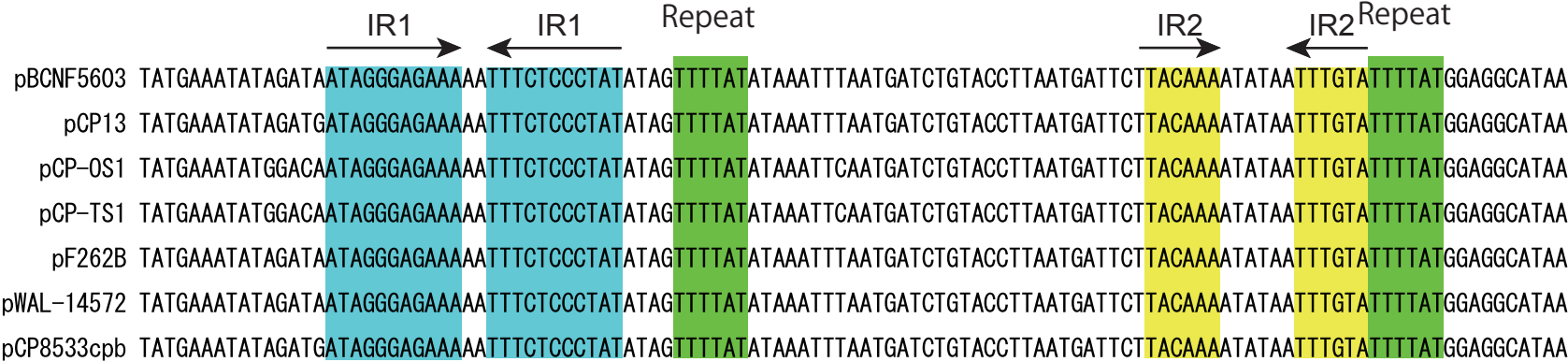

Supplement: Supplementary file 3 — Repeated elements on the putative parS region. The arrow-headed bars depict the two pairs of inverted repeats (IR1 and IR2). And green colored regions depict two short-repeated sequences. [file 12866_2015_443_MOESM3_ESM.pdf]
